# Effect of petroleum-derived substances on life history traits of black bean aphid (*Aphis fabae* Scop.) and on the growth and chemical composition of broad bean

**DOI:** 10.1007/s10646-017-1764-9

**Published:** 2017-01-31

**Authors:** Milena Rusin, Janina Gospodarek, Aleksandra Nadgórska-Socha, Gabriela Barczyk

**Affiliations:** 1 Department of Agricultural Environment Protection, University of Agriculture, al. A. Mickiewicza 21, Krakow, 31-120 Poland; 2 Department of Ecology, University of Silesia, Bankowa 9, Katowice, 40-007 Poland

**Keywords:** Petroleum-derived Substances, *Aphis fabae* Scop., Heavy metals, Macronutrients, Micronutrients, Broad bean

## Abstract

The aim of the study was to determine the effects of various petroleum-derived substances, namely petrol, diesel fuel and spent engine oil, on life history traits and population dynamics of the black bean aphid *Aphis fabae* Scop. and on growth and chemical composition of its host plant *Vicia faba* L. Each substance was tested separately, using two concentrations (9 g kg^−1^ and 18 g kg^−1^). The experiment was conducted in four replications (four pots with five plants in each pot per treatment). Plants were cultivated in both control and contaminated soils. After six weeks from soil contamination and five weeks from sowing the seeds, observations of the effect of petroleum-derived substances on traits of three successive generations of aphids were conducted. Aphids were inoculated separately on leaves using cylindrical cages hermetically closed on both sides. Contamination of aphid occurred through its host plant. Results showed that all tested substances adversely affected *A. fabae* life history traits and population dynamics: extension of the prereproductive period, reduction of fecundity and life span, reduction of the population intrinsic growth rate. In broad bean, leaf, roots, and shoot growth was also impaired in most conditions, whereas nutrient and heavy metal content varied according to substances, their concentration, as well as plant part analysed. Results indicate that soil contamination with petroleum-derived substances entails far-reaching changes not only in organisms directly exposed to these pollutants (plants), but also indirectly in herbivores (aphids) and consequently provides information about potential negative effects on further links of the food chain, i.e., for predators and parasitoids.

## Introduction

Due to their commonness and versatility, petroleum and petroleum-derived substances (PDSs) are used in many industrial fields. However, once they pervade the natural environment, these compounds can adversely affect the growth and development of cultivated plants (Shirdam et al. 2008; Gbadebo and Adenuga 2012). They contribute to the increase of heavy metal content in plants (Onweremadu and Duruigbo 2007; Ujowundu et al. 2011; Rusin et al. 2015), they reduce chlorophyll, protein and carotenoid levels (Achuba 2006; Adenipekun et al. 2008), they constrain plant germination, decrease photosynthetic activity (Agbogidi and Eshegbeyi 2006; Besalatpour et al. 2008; Njoku et al. 2008), and modify the content of micro- and macroelements in plants (Odjegba and Atebe 2007; Shukry et al. 2013). The effect of PDSs on nutrient levels depends chiefly on the type and dose of compounds applied as well as plant species (Wyszkowski and Wyszkowska 2005; Wyszkowski and Ziółkowska 2009a).

Additionally, PDSs modify physicochemical and biological properties of soil, which may also indirectly affect the condition and health status of cultivated plants (Wyszkowska et al. 2002; Lawrence 2013). These substances cause far-reaching changes in the amount and composition of organic content, a reduction of water holding capacity, an increase in the demand for oxygen; they hamper or completely block air transport between the atmosphere and the soil (Caravaca and Rodán 2003; Iturbe et al. 2007), as well as modify the abundance and species composition of edaphic microflora and fauna (Baran et al. 2004). Soil contamination with petroleum causes the sorption complex capacity to drop and reduces the ability to exchange calcium, magnesium, and potassium while also decreasing the availability of these macrocomponents (Agbogidi et al. 2007; Wyszkowski and Ziółkowska 2008). Cultivated plants show varying degrees of susceptibility to the presence of PDSs in soil, the resultant harmful impact depending on numerous factors, such as: type and dose of substances applied, soil properties, soil moisture and pH, oxygen and organic matter content, fertilisation applied and plant species (Wyszkowski and Ziółkowska 2009a).

While the impact of PDSs on plant growth and other organisms directly exposed to the contact with pollutants (e.g., edaphic or aquatic invertebrates) is well documented in scientific literature, information concerning the indirect impact of these compounds from soil via plant on herbivores is still lacking. Arthropods, given their enormous diversity, easy acquisition and breeding, high fecundity rate and short life cycle, are a useful element to consider in a comprehensive assessment of the impact of pollutants on successive trophic chain links. Among these organisms, aphids are highly sensitive to all changes in their food, which quickly occur in their life cycle traits (Harrington and Stork 1995). The changes in biology ensuing from the activity of xenobiotics may, in turn, disturb the synchronisation and occurrence of herbivores and their natural enemies, thus exerting harmful effects on plant production (Percy et al. 2002).

Broad bean (*Vicia faba* L.) is a particularly useful testing plant, both as a bioindicator of oil pollution (Malallah et al. 1996) and for detecting mutagenic substances (Grant et al. 1992, Kanaya et al. 1994), and also due to the fact that aphid life cycle is very fast on this host plant, and hence the impact of the experimental factor can be easily observed.

The soil-plant-herbivore model has been used to date when assessing the environmental impact of pollutants mostly with regard to heavy metals (Merrington et al. 1997a, b, Winder et al. 1999, Green et al. 2006, Kafel et al. 2010) and herbicides (Lipok 2009). In our research, we applied it to determine the effect of PDSs.

The aim of the conducted study was to establish the effect of different concentrations of PDSs, namely, petrol, diesel fuel and spent engine oil on life cycle traits of aphids *A. fabae* feeding on broad beans and on the growth and chemical composition of the host plant.

## Materials and methods

### Experimental setup

The experiment was performed in 2014 in pots that could hold 3.8 kg of soil dry mass. In late April 2014, soil (loamy-sand, pH in KCl = 6.12, pH in H_2_O = 6.98, water-holding capacity = 29.5%, total organic C = 0.97%, N content = 0.09%, available K content = 13.00 mg K_2_O 100 g^−1^, available P content = 11.85 mg P_2_O_5_ 100 g^−1^, Pb content = 25.5 mg kg^−1^, Cd content = 0.99 mg kg^−1^, Ni content = 2.19 mg kg^−1^, Zn content = 51.7 mg kg^−1^, Cu content = 5.02 mg kg^−1^, cation exchange capacity: Ca = 3.71 cmol kg^−1^, Mg = 0.37 cmol kg^−1^, K = 0.33 cmol kg^−1^, Na = 0.01 cmol kg^−1^) was collected from uncultivated areas from level 0–20 cm, finely ground and subsequently spread to form a thin layer on a foil mat. Prior to contamination with PDSs, basal fertilization was applied by treating the soil with 0.27 g N (in form of NH_4_NO_3_), 0.14 g P (in form of KH_2_PO_4_) and 0.21 g K (in form of KCl) per pot. After thoroughly mixing the soil, it was contaminated with engine oil, diesel fuel and petrol at two doses: dose I—9 g of PDS per kg of soil dry mass, dose II—18 g of PDS per kg of soil dry mass. Petrol and diesel fuel came from BP petrol station whereas engine oil from Orlen petrol station. Petrol (BP Unleaded 95) is a complex mixture of volatile hydrocarbons containing paraffins, naphthenes, olefins and aromatic hydrocarbons which contain between C4 and C12 atoms in the molecule (detailed description of the ingredients in Material Safety Data Sheet: http://www.bp.com/content/dam/bp-plus/pl_pl/downloads/PDF/SDS%20benzyna%2095.pdf). Diesel fuel (BP Diesel Fuel) is a mixture of hydrocarbons middle distillates, made between C10 and C28 atoms and may also contain fatty acid methyl ester (FAME) (detailed description in Material Safety Data Sheet: http://www.bp.com/content/dam/bp-plus/pl_pl/downloads/PDF/SDS%20Dieselpdf.pdf). Engine oil (PLATINUM Classic Semisynthetic 10W–40) is a mixture of mineral and synthetic base oils, enriching additives (detailed description in Material Safety Data Sheet: http://www.orlenoil.pl/_layouts/OrlenOilDownload/Download.ashx?downloadUrl=/PL/NaszaOferta/KartyCharakterystyki/KartyCharakterystyki/1117.pdf?) and it was used for one year (in a petrol engine) prior to application in this experiment. Heavy metals content is not mentioned in material safety data sheets of these substances. In order to evenly distribute the contaminants, the predetermined amount of each PDS was poured onto a thinly spread layer of soil on a foil mat using a laboratory pipette. The soil was then thoroughly mixed several times and placed in pots. The area of pot was 706.5 cm^2^ (30 cm in diameter). The pots were arranged so that the distance between them (i.e., external edges) was 50 cm to avoid competition. The non-contaminated soil was placed in identical pots and constituted the control treatment. After one week, broad bean (*Vicia faba* L.) seeds, ‘Bartek’ cultivar, were sown in each pot at an amount of seven seeds per pot. After sprouting, the plants were thinned out and five plants were left in each pot to avoid the competition between germinated seedlings. The distance between plants in each pot obtained in this way amounted approx. 10 cm, corresponding to a standard seed density for this plant under field condition. The pot experiment was performed in quadruplicate.

### Black bean aphid (*Aphis fabae* Scop.) traits and population dynamics

Aphids were inoculated on six week-old plants (30 cm height), using cylindrical cages (12 cm diameter × 20 cm height) made of closely woven airy fabric placed on broad bean leaves (at the same level on each plant to avoid leaf age influence on the pest biology). One cage covered within only one, whole single broad bean leaf used for investigating the life history traits of a single aphid female. The cages were attached to the leaves and hermetically closed on both sides in order to avoid the escape of aphids. The investigations were conducted on *A. fabae* individuals from the own culture of the Agricultural Environment Protection Department maintained on the same host plant, i.e., broad bean, Bartek c.v. Three wingless aphid females were placed in each cage and removed completely once they gave birth to the first larvae. One larva was left in each cage. After it reached sexual maturity, its fecundity was determined every day, while newborn larvae were removed each time except the first one, which was transferred immediately to new cage in order to determine the demographic indicators of subsequent generation. Two cages were placed on each test plant to investigate the life history of the first generation (40 cages in total for treatment) to ensure the right final number of females to study the aphid life history of *A. fabae*. This procedure was applied because some females initially placed on the host plant (using a brush) died before giving birth to the larvae. Cages used to monitor the second and third generation were placed on the subsequent branches of broad bean plants, adopting the rule that it should always be on leaves from the same foliage level of the plant in each treatment and generation analysed. Aphid life span and fecundity were assessed for 25 females of each generation and treatment. The population intrinsic growth rate was calculated using the formula developed by Wyatt and White (1977):

$${r_m} = \frac{{\left( {0.738\, \cdot \,{\rm{ln}}\,{M_d}} \right)}}{d}$$

where:

*r*_*m*_: is the population intrinsic growth rate,
*d*: is the duration of pre-reproductive period (from birth to producing the first offspring),
*M*_*d*_: is the mean number of larvae born in the period from *d* to *2d* days from birth.

The constant value of 0.738 is an approximation of the proportion of the total fecundity produced by a female in the period from *d* to *2d* days from birth.

*A. fabae* is a host-alternating species. Viviparous spring winged or wingless female aphid hatch from diapausing eggs on the primary host. Their winged parthenogenetic descendants disperse to different herbaceous secondary host plants, where numerous parthenogenetic generations take place throughout the growing season. In autumn, decreasing day lengths and temperatures induce the sexual phase of the life cycle. Then winged morphs migrate back to primary hosts, where give birth to sexual females, which mate with the males and deposit overwintering eggs (Sandrock et al. 2011). In our experiment only asexual reproduction of *A. fabae* was investigated.

### Growth of broad bean plants

After the experiment was finished, plants were harvested from the pots and their growth was evaluated in laboratory conditions (total length and mass of shoots, number and mass of leaves, length and mass of roots).

### Chemical composition of plants

In order to determine the nutrients (calcium, potassium, iron, magnesium) and heavy metal (copper, manganese, nickel, lead, zinc, cadmium) concentrations in plants parts, plant material was cleaned of any patches of deposited aphid honeydew and other surface contaminants, washed in tap, next in distilled water. It was then dried at 105 °C for 48 h. A portion of 0.25 g dried plant material was digested with 5 ml of HNO_3_ at 110 °C and then diluted to 10 mL with deionized water. Next, the metal content was measured using flame absorption spectrometry (Unicam 939 Solar) (Azcue and Murdoch 1994; Nadgórska-Socha et al. 2013). The quality of the analytical procedure was checked using a reference material (Certified Reference Material CTA-OTL-1 Oriental Tobacco Leaves) with the same quantities of samples. Carbon, nitrogen and sulphur contents were determined in a Variomax CNS analyzer. Due to the small amount of plant material obtained for chemical analyses, no assays for nitrogen, carbon and sulphur levels were conducted on plant roots from samples contaminated with diesel fuel at both doses and with petrol at the dose of 18 g kg^−1^. The following indicators were calculated: K/(Ca+Mg), Ca/Mg, N/S, representing ratios between selected nutrients in plants.

### Statistical analysis

The obtained results were analyzed, checked for normality (Shapiro–Wilk test with Lilliefors correction) and equality of variance (Levene’s test) and when necessary the data were log transformed. The significance of differences between the means were tested by one-factor variance analysis (STATISTICA 10.0 software), and the means were differentiated by Fisher’s LSD test at *p* < 0.05.

Multiple regression equations were derived to determine which of the PDSs dose, accumulated heavy metal and nutrient contents influenced aphid traits. The method of stepwise forward regression was applied. The equations concerned the relationships between life span, fecundity as well as intrinsic growth rate and the examined of PDSs dose (9 g kg^−1^ and 18 g kg^−1^ respectively), accumulated nutrients (calcium, potassium, iron, magnesium) and heavy metals: copper, manganese, nickel, lead, zinc, cadmium. Significance of differences was set at a level of *p* < 0.05. Moreover, the value of the linear correlation coefficient between fecundity and life span was calculated.

CANOCO 4.5 was used to carry out Principal Component Analysis (PCA) (Ter Braak and Šmilauer 2002). Principal Component Analysis assessed the relationships between plant composition and PDS contamination.

## Results

### Life cycle traits of black bean aphid (*Aphis fabae* Scop.)

**Fig. 1:**
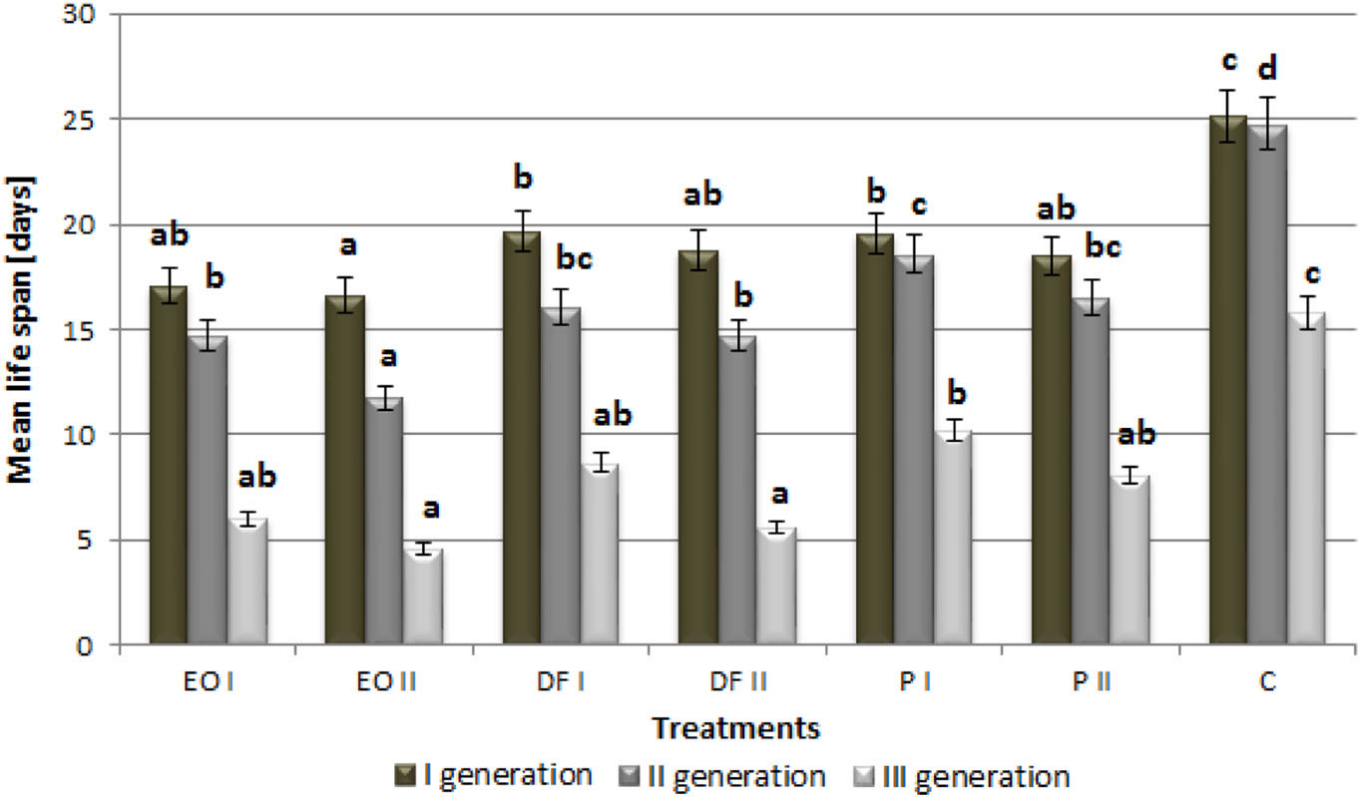
The effect of petroleum-derived substances on mean life span of *Aphis fabae* Scop. (days). *C* control soil, *P* soil contaminated with petrol, *EO* soil contaminated with engine oil, *DF* soil contaminated with diesel fuel, *I, II* doses of pollutants. Values marked by different letters for each generation are statistically different (*p* < 0.05)

**Fig. 2:**
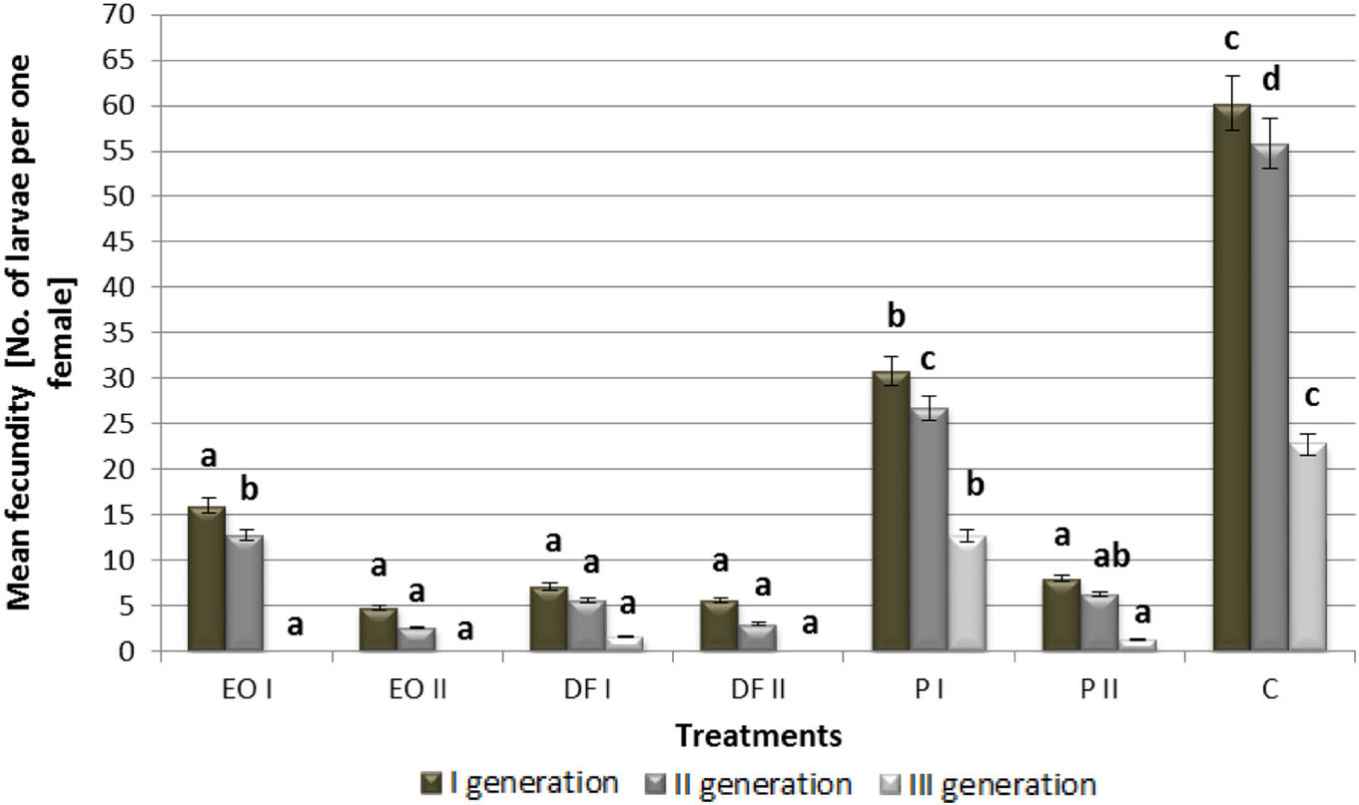
The effect of petroleum-derived substances on mean fecundity of *Aphis fabae* Scop. (No. of larvae per one female). Symbols as in Fig. 1. Values marked by different letters for each generation are statistically different (*p* < 0.05)

**Fig. 3:**
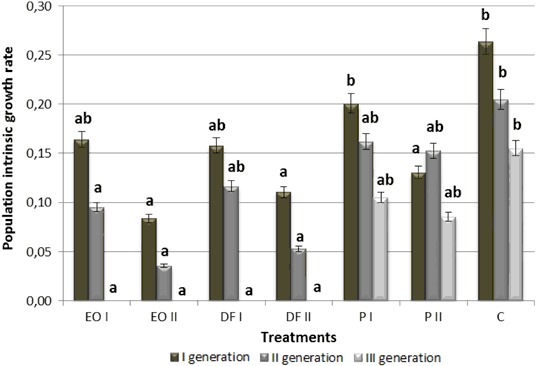
The effect of petroleum-derived substances on population intrinsic growth rate (*r*_*m*_) of *Aphis fabae* Scop. Symbols as in Fig. 1. Values marked by different letters for each generation are statistically different (*p* < 0.05)

All PDSs, at both doses used, caused a significant life span reduction in the three generations of *A. fabae* that feed on plants growing in contaminated soil relative to control conditions (by approx. 30%) (Fig. 1). As the content of xenobiotics in the soil increased, most frequently the black bean aphid life span declined. Moreover aphid life span decreased over time in subsequently emerging generations of the pest under all conditions (including control). All PDSs resulted in a significant drop of fecundity in all generations of *A. fabae* females, at both doses applied (Fig. 2). The most adverse effect on this parameter was exerted by engine oil and diesel fuel at the dose of 18 g kg^−1^, which caused female fecundity in the first and second generation to decrease by over 90% and completely prevented the birth of new larvae in the third generation. As in the previous case analysed, aphid fecundity increasingly declined in subsequent generations. The duration of pre-reproductive period (*d*) of *A. fabae* ranged from 9.3 to 12.0 days for the first generation, from 9.0 to 11.9 days for the second one and from 11.3 to 12.8 for the third one (Table 1). Both doses of engine oil, as well as 18 g kg^−1^ of diesel fuel caused the process of birthing larvae to cease completely in the third generation of females. All PDSs applied at both doses (except the lower dose of engine oil) resulted in a significant elongation of the pre-reproductive period in the first two generations of females. *M*_*d*_ in all generations was the largest in the controls (Table 1). In aphids living on plants contaminated by engine oil and diesel fuel, females in all generations gave birth to significantly fewer larvae vs. control during the evaluated period (*M*_*d*_). Petrol at the dose of 9 g kg^−1^ led to a significant decrease in *M*_*d*_ in the third generation of aphids, while the dose of 18 g kg^−1^ produced this effect both in the first and third generation. The population intrinsic growth rate reached the highest values in the first generation of the pest and dwindled in next generations, being reduced to zero under engine oil and diesel fuel contamination (Fig. 3). In the first generation, only the higher doses of PDSs (18 g kg^−1^) induced a significant drop in the population intrinsic growth rate relative to the control. However, both engine oil at the two doses and diesel fuel at the higher dose caused the studied parameter to fall significantly in the second generation. A significant reduction of the analysed parameter in the third generation was observed in females that feed on plants growing in the soil treated with engine oil and diesel fuel, both at the dose of 9 g kg^−1^ and 18 gkg^−1^.

**Table 1:** The effect of petroleum-derived substances on some biological parameters

Details	*d*			*M*_*d*_		
Generation	I	II	III	I	II	III
EO I	10.0^ab^*	10.8^ab^	–	9.3^ab^	4.1^ab^	0^a^
EO II	10.8^bc^	11.1^b^	–	3.4^a^	1.7^a^	0^a^
DF I	12.0^c^	11.9^c^	12.8^b^	13.1^b^	6.5^ab^	1.0^a^
DF II	11.3^bc^	11.7^bc^	–	5.4^a^	2.3^a^	0^a^
P I	10.8^bc^	10.3^ab^	11.5^a^	18.8^bc^	9.7^abc^	5.2^b^
P II	11.5^c^	11.7^bc^	12.2^ab^	7.6^ab^	11.2^bc^	4.1^b^
C	9.3^a^	9.0^a^	11.3^a^	27.8^c^	12.2^c^	10.8^c^

*d* duration of pre-reproductive period, *M*_*d*_ mean number of larvae born in time = d of *Aphis fabae* Scop

^*^Means in columns marked with the same letters do not differ significantly according to LSD test at *p* < 0.05. Symbols as in Fig. 1

### Growth of broad bean plants

All PDSs applied (except petrol at the dose of 9 g kg^−1^) had a negative impact on the growth of broad bean plants, significantly reducing the length and mass of shoots and roots, as well as the number and mass of leaves (Table 2). However, 9 g kg^−1^ of petrol caused a significant decrease in root length and mass. The growing dose of each xenobiotic was usually mirrored by the increasingly harmful effect on the growth of plant.

**Table 2:** The effect of petroleum-derived substances on the growth of *Vicia faba* L

Details	Sum of shoots length per plant (cm)	Mass of shoots per plant (g)	Number of leaves per plant (pcs.)	Mass of leaves per plant (g)	Root length per plant (cm)	Mass of root per plant (g)
EO I	31.07^a*^	11.94^a^	31.93^b^	13.71^b^	3.87^a^	6.0^a^
EO II	25.13^a^	9.12^a^	22.13^a^	9.35^a^	3.63^a^	5.53^a^
DF I	28.36^a^	11.60^a^	28.18^ab^	11.41^ab^	3.14^a^	4.85^a^
DF II	25.88^a^	9.94^a^	23.75^ab^	10.02^ab^	3.38^a^	5.18^a^
P I	48.13^b^	18.75^b^	56.93^c^	24.54^c^	7.60^b^	12.06^b^
P II	25.22^a^	9.72^a^	19.11^a^	8.24^a^	4.22^a^	6.54^a^
C	55.33^b^	21.63^b^	64.13^c^	27.68^c^	10.00^c^	15.88^c^

^*^Means in columns marked with the same letters do not differ significantly according to LSD test at *p* < 0.05. Symbols as in Fig. 1

### Plant components and heavy metal content

The impact of PDSs on the mineral nutrient content in plants was varied and depended on the analysed component, the dose and type of contaminant, as well as plant part. All PDSs caused a significant rise in the sulphur content in broad bean leaves and shoots, and usually also a rise in carbon content in these organs. Engine oil caused nitrogen content in plant leaves to lower significantly, decreasing also calcium content in leaves and shoots. Both applied doses of diesel fuel resulted in a significant decrease in potassium and nitrogen content in the leaves. On the other hand, they induced an increase in magnesium content in the leaves and shoots of the plants. Petrol led to an elevated level of nitrogen in broad bean leaves and shoots but also reduced level of calcium in these organs. Detailed data are provided in Supplemental Tables S1 and S2.

All PDSs most frequently caused a significant decrease in the computed values of Ca/Mg and N/S ratios in plant leaves and shoots (Table 3). Engine oil at both doses and diesel fuel at 9 g kg^−1^ induced an increase in Ca/Mg ratio in plant roots. Diesel fuel significantly decreased K/(Ca+Mg) ratio in all plant organs analysed. Both doses of engine oil augmented K/(Ca+Mg) ratio in broad bean leaves while reducing it in roots. The lower dose of this contaminant also caused this indicator to rise in plant shoots. Petrol increased K/(Ca+Mg) ratio in plant roots, whereas the dose of 18 g kg^−1^ additionally increased this indicator in plant leaves while reducing it in shoots.

**Table 3:** The effect of petroleum-derived substances on the ratio of nutrients in *Vicia faba* L

Details	K/(Ca+Mg)	Ca/Mg	N/S
Leaves			
EO I	1.66^de*^	4.78^b^	13.60^b^
EO II	1.75^e^	3.67^a^	12.41^a^
DF I	0.67^a^	5.81^c^	12.19^a^
DF II	0.86^a^	6.33^cd^	13.51^b^
P I	1.27^bc^	7.27^de^	16.91^d^
P II	1.48^cd^	4.52^b^	14.60^c^
C	1.24^b^	7.35^e^	18.92^e^
Shoots			
EO I	8.46^d^	4.02^a^	8.25^a^
EO II	7.21^c^	3.81^a^	9.93^b^
DF I	3.87^a^	4.66^bc^	7.98^a^
DF II	3.74^a^	4.43^b^	7.42^a^
P I	7.45^c^	5.00^c^	11.95^c^
P II	6.31^b^	3.88^a^	14.17^e^
C	7.11^c^	6.32^d^	13.10^d^
Roots			
EO I	2.72^c^	6.66^c^	6.87^a^
EO II	1.02^a^	6.54^c^	7.43^a^
DF I	1.43^b^	6.67^c^	–
DF II	2.51^c^	4.05^a^	–
P I	5.09^f^	4.87^ab^	9.17^a^
P II	4.57^e^	5.18^ab^	–
C	3.79^d^	5.42^b^	9.05^a^

^*^Means in columns for each organ of plant marked with the same letters do not differ significantly according to LSD test at *p* < 0.05. Symbols as in Fig. 1

As was the case with macrocomponents, the content of heavy metals in plants resulting from the presence of PDSs in soil was diverse (Table 4). All substances caused zinc and cadmium levels in plant leaves and roots as well as nickel level in shoots to drop. Both doses of engine oil also triggered a fall in the manganese content of broad bean leaves and shoots. However, they increased copper content in plant roots. Diesel fuel generated an increase in manganese level in broad bean leaves and shoots and a decrease in nickel level in roots. The diesel fuel dose of 9 g kg^−1^ caused an elevated lead content in leaves and increased root levels of copper and manganese. Both doses of petrol decreased shoot and leaf levels of manganese, whereas they increased shoot levels of lead.

**Table 4:** The effect of petroleum-derived substances on content of selected heavy metals in *Vicia faba* L. (mg kg^−1^)

Details	Cu	Mn	Ni	Pb	Zn	Cd
Leaves						
EO I	20.21^a*^	377.32^a^	3.76^bc^	17.89^a^	226.18^b^	1.94^ab^
EO II	19.78^a^	474.22^b^	3.84^bc^	30.40^ab^	223.93^b^	1.80^a^
DF I	19.11^a^	879.84^e^	3.06^ab^	42.73^b^	225.44^b^	2.37^c^
DF II	17.82^a^	784.97^d^	2.32^a^	14.78^a^	222.75^b^	2.02^ab^
P I	20.63^a^	317.69^a^	4.62^c^	12.87^a^	201.80^a^	2.13^bc^
P II	23.64^b^	354.61^a^	2.77^a^	30.76^ab^	214.94^ab^	2.16^bc^
C	18.69^a^	580.96^c^	3.16^ab^	9.91^a^	257.95^c^	3.17^d^
Shoots						
EO I	12.87^cd^	82.72^b^	1.24^a^	8.73^a^	138.53^a^	1.55^a^
EO II	10.00^ab^	91.90^b^	1.24^a^	9.91^ab^	148.47^ab^	1.79^a^
DF I	9.18^a^	132.83^d^	1.20^a^	6.88^a^	152.20^ab^	1.83^a^
DF II	11.12^abc^	129.97^d^	1.30^a^	13.72^b^	161.53^b^	1.84^a^
P I	14.70^de^	49.72^a^	1.50^a^	32.18^c^	154.29^ab^	1.99^a^
P II	16.12^e^	56.56^a^	1.39^a^	34.73^c^	167.72^b^	1.89^a^
C	12.26^bcd^	113.34^c^	2.00^b^	13.06^b^	137.07^a^	2.19^a^
Roots						
EO I	39.33^b^	481.37^cd^	1.97^bc^	20.53^a^	272.78^c^	4.14^b^
EO II	38.63^b^	583.72^e^	1.61^ab^	21.70^ab^	265.64^c^	3.86^ab^
DF I	37.31^b^	558.11^de^	1.41^a^	23.20^ab^	224.39^b^	4.56^b^
DF II	29.16^a^	427.90^bc^	1.72^ab^	20.84^a^	166.78^a^	3.16^a^
P I	27.20^a^	200.69^a^	2.39^d^	23.77^ab^	230.69^b^	6.20^c^
P II	35.32^b^	388.63^b^	2.46^d^	24.91^ab^	262.75^c^	4.46^b^
C	24.72^a^	428.26^bc^	2.35^cd^	27.80^b^	357.54^d^	7.83^d^

*Means in columns for each organ of plant marked with the same letters do not differ significantly according to LSD test at *p* < 0.05. Symbols as in Fig. 1

### Relationships between black bean aphid traits, soil contamination with PDSs and broad bean chemical composition

Multiple regression revealed that PDSs and Zn had a negative effect on life span of aphids (Table 5). PDSs and S and Mn content had a negative effect on fecundity and intrinsic growth rate (S and Mn only for fecundity). Cadmium (for both above mentioned traits) and N, K and Ca (for intrinsic growth rate) had a positive effect. Fecundity of aphids was strongly correlated with its life span (the correlation value amounted 0.892).

**Table 5:** Multiple regression equations (*p* < 0.05)

							*R* ^2^
Life span = 9.03 −0.36(ps dose) + 0.84(Cd) −0.46(Zn)							0.892
(2.87) (0.15) (0.19) (0.15)							
Fecundity = 32.65 −0.36(ps dose) −0.46(S) –0.45(Mn) + 0.428(Cd)							0.978
(8.98) (0.07) (0.06) (0.06) (0.07)							
Intrinsic growth rate = −0.23 −0.54(ps dose) + 0.757(N) + 1.00(K) + 0.585(Ca)							0.921
(0.09) (0.15) (0.12) (0.26) (0.23)							

**Fig. 4:**
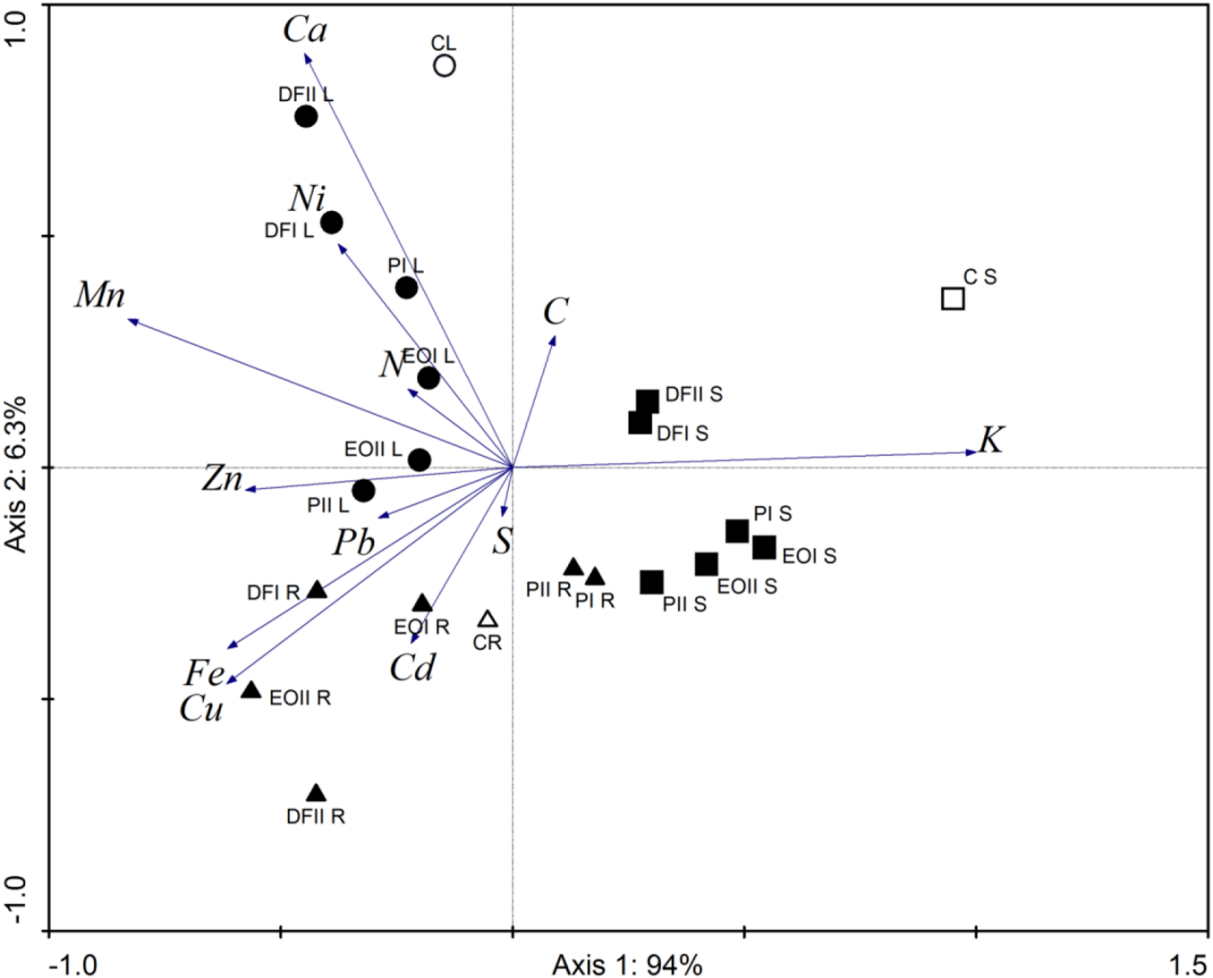
Principal component analysis of element levels in *V. faba* in organs, and soil contamination with petroleum-derived substances. Symbols as in Fig. 1. *L* leaves, *S* shoots, *R* roots

Principal component analysis of element levels in *V. faba* organs, and soil contamination with PDSs showed that the elements correlated with the 1st ordination axis accounting for more than 93% of variation of the analyzed samples (Fig. 4). K and Zn contents were the most correlated with the 1st ordination axis. The highest concentrations of Ca, Ni, and Mn were found in plants exposed to diesel fuel and engine oil in both doses and petrol at the dose of 9 g kg^−1^, as well as in leaves from control plants. In the roots the highest content of Pb and Cd were found in plants planted in soil contaminated with diesel fuel and engine oil. Also Fe and Cu levels were the highest in plants from the soil contaminated with diesel fuel at the dose of 9 g kg^−1^ and engine oil at the dose of 18 g kg^−1^. The lowest content of the investigated elements except K was found in shoots of broad been.

## Discussion

In conducted experiment only the parthenogenetic part of the life cycle of *A. fabae* was investigated. The effects of moving to other host plants (possibly uncontaminated) and of sexual reproduction were not accounted. Therefore, the experiment does not provide a complete response on the effect of PDSs on *A. fabae* biology. However, given that on herbaceous plants can develop to 10 asexual generations (which comprises about 80% of full life cycle), this may involve significant implications both for the whole pest population in a given area, and for its natural enemies.

The average fecundity of wingless females of *A. fabae* on broad bean in uncontaminated conditions ranges from 15.3 to 59.2 larvae and depends on the cultivars of broad bean and the generation of pests. It usually declines in subsequent generations, what is the result of changes in the host plant, associated with its growth and ageing (Cichocka et al. 2002). Aphid life span is about 23 days (also usually decreases across generation) and intrinsic growth rate amounts to 0.345 (Douglas 1997). In our experiment analysed life history traits were at a similar level, while population intrinsic growth rate was lower and ranged 0.156–0.264 depending on generation. All PDSs used in this experiment had an adverse impact on the life cycle traits of black bean aphid, causing its fecundity to decline, shortening its life span, prolonging the pre-reproductive period, and usually causing the population intrinsic growth rate to drop. Available scientific literature lacks information concerning the effect of soil contamination with PDSs on the life cycle traits of herbivores. Causes of the noted changes in black bean aphid life history can be partly deduced by confronting the obtained results with the changes of chemical composition in the host plant ensuing from soil contamination, as well as with the results provided by studies concerning other types of soil contamination, e.g., with heavy metals, especially in view of the known fact that PDSs cause heavy metal levels to increase in soil (Okonokhua et al. 2007; Ujowundu et al. 2011; Wyszkowski and Sivitskaya 2012).

Changes in life history traits of aphids caused by the presence of PDSs in the soil may result from impairment of the host plant trophic value. PDSs impede the growth and development of crops and alter their macro- and microcomponent contents. All PDSs used in the performed experiment had a negative impact on the growth of broad bean plants and this impact usually intensified in a dose-dependent manner. Many authors accentuate the harmful effect of PDSs on crop growth and development (Liste and Felgentreu 2006; Njoku et al. 2008; Njoku et al. 2012; Osuagwu et al. 2013; Lopes and Piedade 2014; Rusin et al. 2015). The adverse action of PDSs on cultivated plants may result from the lowered content of nutrients available to plants consequent upon the presence of these contaminants in soil. Disruption in photosynthesis and decreased chlorophyll levels may cause plant growth inhibition or death (Odjegba and Atebe 2007). These substances increase soil density, which causes soil pores to clog and consequently alters the physical, chemical and biological properties of soil and disturbs water and nutrient uptake by plants. PDSs may also block the molecular transport in plant cells, which may contribute to the limited development of vegetative and generative organs (Osuagwu et al. 2013). Aqueel et al. (2014) confirmed that plant quality can affect the feeding of aphids and other consumers with higher trophic level. On high quality plants the aphids produced most offspring and were characterized by a higher population intrinsic growth rate (Stadler et al. 2002). Bad condition of plants, demonstrated by a significant growth restriction, caused by the presence of heavy metals in the soil, reduces fecundity of *A. fabae* and results in a shortening of its life span (Gospodarek 2012).

Aphids feed by piercing the phloem of their food plant and are very sensitive to changes in plant quality (Omacini et al. 2001). Quality of plants is largely determined by the content of macro- and micronutrients which directly affect potential and achieved herbivore fecundity (Awmack and Leather 2002). PDSs used in this experiment most often caused carbon and sulphur levels to rise in plant leaves and shoots, but both diesel fuel and engine oil contributed to a drop in leaf nitrogen level. Rusin et al. (2015) also showed that PDSs reduced nitrogen level in the leaves of broad bean, but some of them caused a reduction in sulphur and carbon content, which only partially corresponds to results of the present experiment. The discrepancies may be due to different doses of pollutant used in the two experiments. In soils contaminated with PDSs, the nitrogen-to-carbon ratio is distorted by the presence of hydrocarbons. This contributes to many nitrogen reactions being curbed in soil and also lowers the intensity of ammonification and nitrification processes (Adam and Duncan 2003). PDSs provide an increased carbon content in soil, which results from their structure (they contain aliphatic hydrocarbons, cycloalkanes, olefins and arenes) (Riffaldi et al. 2006; Wyszkowski and Sivitskaya 2012). Moubasher et al. (2015) showed that petroleum-derived hydrocarbons usually caused no significant change in the sulphur content of *Bassia scoparia* (L.) roots and shoots. In our experiment, PDSs caused a significant increase in the content of this nutrient in broad bean leaves and shoots. Again, the disparities may stem from the different species of test plant and a different dose of substances applied in the two experiments. The impact of PDSs on the content of calcium, magnesium, potassium and iron in plants was diverse and depended on the analysed component, dose and type of contaminant and part of plants. PDSs can have various effects on plant Ca, Mg, K and Fe content, depending on the species, doses and the part of the plant (Wyszkowski and Ziółkowska 2009a, b). An optimum value of K/(Ca+Mg) ratio for plants to grow and develop should range within 1.6/1–2.1/1 (Matraszek et al. 2015). The effect of PDSs on the value of the said indicator was varied and depended on the substance applied, its dose, as well as plant part examined. The Ca/Mg value in our experiment ranged within 3.67–7.35. PDSs usually acted towards a decrease in the value of this indicator in leaves and shoots. The N/S ratio informs of the degree to which plants are supplied with sulphur and its value in vegetative plant organs should range around 15/1 (Jamal et al. 2010). In the conducted experiment, the value of this indicator was most often slightly below its optimum, and it was also noted that PDSs usually decreased it significantly in broad bean leaves and shoots. These changes in nutrients content and nutrients ratio may result in a deteriorated fodder quality for pests (Nadgórska-Socha et al. 2005), which can explain the negative impact of PDSs on life history traits of the *A. fabae*. Numerous authors indicate that the increasing nitrogen content in host plants entails enhanced infestation by aphids (Honêk and Martinkova 2002; Davies et al. 2004; Naluyange et al. 2014). In this experiment, engine oil and diesel fuel caused a significant decrease in the nitrogen content of broad bean leaves when applied at both doses, which may have conducted to life span shortening and fecundity reduction in all three black bean aphid generations. Jansson and Ekbom (2002) also found that higher content of N and K in petunia plants accelerates the development of *Macrosiphum euphorbiae* and has a positive effect on its life span. In our experiment PDSs often decreased the content of potassium in shoots and leaves of broad bean which may adversely affect life history traits of *A. fabae*. Moreover, PDSs usually contributed to a reduction in calcium content. Changes in the content of this macrocomponent may also cause disturbances in the occurrence of the piercing-sucking herbivores (Sądej and Sądej 2001). Available scientific literature lacks information concerning the effect of other analysed nutrients content on life history traits of aphids.

The composition of PDSs contains heavy metals, polycyclic aromatic hydrocarbons (PAHs) and other chemical admixtures, which are toxic to living organisms. Therefore, the adverse effect of PDSs on life history traits of aphids may also result from the transfer of harmful substances from the soil through plants to the aphids. Merrington et al. (2001) found that aphids accumulate more toxic metals in the bodies than their host plants in the tissues. Numerous authors underscore that soil contamination with heavy metals may strongly affect the life history traits of aphids (Gospodarek 2005, 2012; Görür 2007, 2009). Davies et al. (1998) found that black bean aphid larvae reach sexual maturity after 5.85 ± 0.35 days under laboratory conditions, whereas Gospodarek (2012) demonstrated that this period may extend to 12.2 days in conditions of soil contamination with heavy metals. The PDSs in the present experiment also caused elongation of the pre-reproductive period that reached up to 12.8 days, while the control values ranged between 9.0 and 11.3 days. Soil pollution with heavy metals such as Zn and Ni used separately reduces fecundity and life span of *A. fabae*, 2–3 times and by approx. 30% respectively (Gospodarek 2012). In our experiment fecundity of aphids under the influence of lower PDSs doses decreased 2–10 times, while in the case of higher doses—10–20 times and life span decreased by approx. 30–50% on average. Nevertheless, it should be noted that in conducted by mentioned author studies, the content of Zn and Ni in broad bean tissues was several dozen times higher than in our experiment. In mentioned experiment (Gospodarek 2012) an increase in Pb, Cd and Cu content in broad bean tissues did not contribute to a reduction in fecundity and life span of *A. fabae*. In our experiment heavy metals content in leaves and shoots of broad bean plants was varied depending on the type of pollutants, its dose and type of metal.

PDSs alter heavy metal levels in soil, which may also indirectly affect these levels in plant organs (Santos-Echeandia et al. 2008). Some authors have shown that PDSs cause soil levels of cadmium, lead, copper and manganese to rise (Okonokhua et al. 2007; Ujowundu et al. 2011; Wyszkowski and Sivitskaya 2012). It can explain the elevated content of Cu in broad bean roots in the present experiment. However, all PDSs caused a drop in cadmium content of broad bean leaves and roots. Rusin et al. (2015) also demonstrated that spent engine oil and petrol contribute to a significant reduction of cadmium levels in broad bean leaves. Similar findings were made by Nwaichi et al. (2014), who showed that PDSs cause the level of this metal to decrease in the leaves of *Vernonia amygdelina* i *Talinum triangulare*. The authors also showed that PDSs increased lead levels in plant leaves, which was confirmed in the study by Rusin et al. (2015). In the present experiment, only diesel fuel at the dose of 9 g kg^−1^ induced lead level to rise in broad bean leaves. Petrol also caused the content of this metal to grow in shoots, though shoot and root lead contents were observed to fall as a result of engine oil and diesel fuel present in soil.

Available scientific literature provides scarce information about the transfer of PAHs from contaminated soil to plants. Gao and Zhu (2004) found that accumulation of phenanthrene and pyrene in plants was elevated with the increase of their soil concentrations. This indicates that adverse effect of PDSs on life history traits of *A. fabae* may result not only from the transfer of heavy metals but also other harmful compounds of PDSs such as PAHs.

The magnitude of changes in the content of macro and micronutrients as well as heavy metals content (when we consider them separately) was not so great to make conclusion that these changes are the main causes of adverse effect of PDSs on life history traits of *A. fabae*. This suggests the possibility of a synergistic effect of impairment of the plant trophic value and the transfer of toxic substances in this soil-plant-aphids interaction.

## Conclusions

1. All PDSs used had an adverse effect on life history traits of black bean aphid, causing a decline in its fecundity, shortening its mean life span, extending the pre-reproductive period, and most often lowering the population intrinsic growth rate.
2. The adverse effect of PDSs on life history traits of aphids may be associated with synergistic influence of impairment of the host plant trophic value and the transfer of toxic substances from the soil through plants to the aphids. PDSs produced a negative effect on the growth of broad bean plants and modified nutrients content. These substances most frequently caused an increase in carbon and sulphur levels in plant leaves and shoots, though both diesel fuel and engine oil caused a decrease in nitrogen level in broad bean leaves. All substances applied caused a decrease in iron and potassium levels in plant shoots, as well as most frequently a decrease of calcium level in leaves. Diesel fuel caused an increase in manganese levels in broad bean leaves and shoots, engine oil—an increase in copper levels in roots, while petrol—an increase in lead levels in plant shoots.
3. Obtained results indicate that soil contamination with PDSs entails far-reaching changes not only in organisms directly exposed to these pollutants (plants), but also indirectly in herbivores (aphids) and consequently provides information about potential negative effects on further links of the food chain, i.e., for predators and parasitoids.

## Electronic supplementary material

Supplementary Information
