# Supplementary material for: Effect of petroleum-derived substances on life history traits of black bean aphid (Aphis fabae Scop.) and on the growth and chemical composition of broad bean
Source: Ecotoxicology. 2017 Jan 31;26(3):308–19. doi: 10.1007/s10646-017-1764-9 (PMC5397440; doi:10.1007/s10646-017-1764-9)
Supplement: Supplementary file 1 — Supplementary Information [file 10646_2017_1764_MOESM1_ESM.docx]

**Supplemental Information**

**Effect of petroleum-derived substances on life history traits of black bean aphid (*Aphis fabae* Scop.) and on the growth and chemical composition of broad bean**

Milena Rusin^1^, Janina Gospodarek^1^, Aleksandra Nadgórska-Socha^2^, Gabriela Barczyk^2^

^1^Department of Agricultural Environment Protection, University of Agriculture, al. A. Mickiewicza 21, 31-120 Krakow, Poland,

^2^Department of Ecology, University of Silesia, Bankowa 9, 40-007 Katowice, Poland

**Supplemental Table S1:** The effect of petroleum-derived substances on content of selected macronutrients in *Vicia faba* L. (g kg^-1^). C – control soil, P – soil contaminated with petrol, EO – soil contaminated with engine oil, DF – soil contaminated with diesel fuel, I, II – doses of pollutants. *Means in columns for each organ of plant marked with the same letters do not differ significantly according to LSD test at p < 0.05.

| **Details** | **N** | **C** | **S** |
| --- | --- | --- | --- |
| **Leaves** | | | |
| EO I | 48.17^b*^ | 372.93^de^ | 3.54^c^ |
| EO II | 43.79^a^ | 375.91^e^ | 3.53^c^ |
| DF I | 49.57^c^ | 365.87^c^ | 4.07^d^ |
| DF II | 43.81^a^ | 362.62^bc^ | 3.24^b^ |
| P I | 58.47^e^ | 370.55^d^ | 3.46^c^ |
| P II | 64.88^f^ | 353.99^a^ | 4.45^e^ |
| C | 50.37^d^ | 359.96^b^ | 2.66^a^ |
| **Shoots** | | | |
| EO I | 35.03^a^ | 369.44^c^ | 4.25^c^ |
| EO II | 43.04^b^ | 370.94^c^ | 4.33^c^ |
| DF I | 40.94^b^ | 364.04^ab^ | 5.14^e^ |
| DF II | 34.97^a^ | 368.82^bc^ | 4.71^d^ |
| P I | 42.79^b^ | 368.08^bc^ | 3.59^b^ |
| P II | 61.02^c^ | 362.23^a^ | 4.31^c^ |
| C | 33.08^a^ | 360.17^a^ | 2.53^a^ |
| **Roots** | | | |
| EO I | 38.04^a^ | 349.40^a^ | 3.95^a^ |
| EO II | 35.00^a^ | 349.05^a^ | 4.98^a^ |
| DF I | - | - | - |
| DF II | - | - | - |
| P I | 45.61^b^ | 338.21^a^ | 5.54^a^ |
| P II | - | - | - |
| C | 35.59^a^ | 306.19^a^ | 4.71^a^ |

**Supplemental Table S2:** The effect of petroleum-derived substances on content of selected nutrients in *Vicia faba* L. (mg kg^-1^). *Means in columns for each organ of plant marked with the same letters do not differ significantly according to LSD test at p < 0.05. Symbols as in Table S1.

| **Details** | **Ca** | **K** | **Fe** | **Mg** |
| --- | --- | --- | --- | --- |
| **Leaves** | | | | |
| EO I | 15167.06^a*^ | 30289.12^cd^ | 1121.00^b^ | 3170.77^b^ |
| EO II | 13071.00^a^ | 29015.52^cd^ | 938.78^ab^ | 3564.62^c^ |
| DF I | 20618.49^b^ | 20751.03^ab^ | 973.53^ab^ | 3552.33^c^ |
| DF II | 23801.33^c^ | 18450.05^a^ | 780.99^a^ | 3767.20^c^ |
| P I | 19183.96^b^ | 27804.71^cd^ | 1382.12^c^ | 2645.80^a^ |
| P II | 13602.93^a^ | 24676.52^bc^ | 1345.70^c^ | 3015.92^b^ |
| C | 23351.79^c^ | 32998.22^d^ | 1043.78^b^ | 3185.13^b^ |
| **Shoots** | | | | |
| EO I | 6106.03a | 64479.23^c^ | 96.70^a^ | 1519.68^ab^ |
| EO II | 6418.62^a^ | 58438.45^b^ | 148.55^b^ | 1688.21^c^ |
| DF I | 11107.89^bc^ | 52075.30^a^ | 115.14^ab^ | 2385.21^d^ |
| DF II | 11575.07^c^ | 52878.88^a^ | 105.04^a^ | 2611.84^e^ |
| P I | 6911.58^a^ | 61739.07^bc^ | 235.63^c^ | 1383.04^a^ |
| P II | 6667.75^a^ | 52699.71^a^ | 228.61^c^ | 1721.39^c^ |
| C | 10316.22^b^ | 84955.79^d^ | 358.92^d^ | 1634.07^bc^ |
| **Roots** | | | | |
| EO I | 9233.46^c^ | 28873.84^c^ | 2352.68^c^ | 1399.36^ab^ |
| EO II | 9160.31^c^ | 10800.29^a^ | 2466.95^c^ | 1407.93^ab^ |
| DF I | 10991.07^d^ | 17987.84^b^ | 2519.63^c^ | 1646.84^c^ |
| DF II | 5484.42^a^ | 17163.04^b^ | 1690.83^a^ | 1355.63^a^ |
| P I | 7634.40^b^ | 46874.22^e^ | 2037.55^b^ | 1569.96^bc^ |
| P II | 8191.54^b^ | 44702.76^e^ | 2353.05^c^ | 1587.08^bc^ |
| C | 7928.95^b^ | 35604.99^d^ | 2322.53^c^ | 1465.06^abc^ |
